# The Structure of Vertebrate Antigen Receptor Repertoires: An Evolutionary Perspective

**DOI:** 10.1111/imr.70154

**Published:** 2026-08-03

**Authors:** Thomas Boehm, Orlando B. Giorgetti

**Affiliations:** ^1^ Max Planck Institute of Immunobiology and Epigenetics Freiburg Germany; ^2^ Institute for Immunodeficiency, Center for Chronic Immunodeficiency, University Medical Center Freiburg Germany; ^3^ Max Planck Institute for Biology Tübingen Germany

**Keywords:** BCR, cytidine deaminase, somatic diversification, TCR, TdT, VLR

## Abstract

In vertebrate adaptive immune systems, somatically diversified antigen receptors assume a central role in self/nonself discrimination. Attesting to the presence of a unique but unknown selective environment at early stages of vertebrate evolution, this facility emerged twice, in the ancestors of jawless and jawed vertebrates. Thus, the molecular structure of incomplete antigen receptor genes and their mode of assembly into functional genes are different in the two sister groups of vertebrates. It appears that adaptive immunity evolved in steps, trading immunologically favorable diversity of antigen receptor repertoires against the inherent risks of potentially destructive self recognition. Initially, the associated quality control mechanisms were largely cell‐autonomous and grounded in the evolutionarily selected sequence composition of individual components available for assembly. At later stages, diversity increased in lock‐step with emerging cell‐nonautonomous quality control strategies: primary lymphoid organs spatially and temporally coupled repertoire development and assessment for self reactivity; regulatory cell types emerged to keep self reactive clones in check in the periphery. In this review, we discuss how comparative studies of vertebrate species situated at key positions in the phylogenetic tree have revealed traces of the evolutionary past of adaptive immune systems.

## 1 Introduction

About 550 million years ago, early vertebrates evolved a radically different form of animal immune system. This involved the emergence of novel cell lineages, such as lymphocytes; tissue types, such as primary lymphoid organs; and genome modification by cytidine deaminases and RAG recombinases in a cell type‐specific manner. In contrast to ancient innate immune facilities that are based on germline‐encoded components, the clonal representation of a somatically generated, structurally diverse repertoire of antigen receptors emerged as the key characteristic of this revolutionary type of animal immunity. With time, ancient innate and novel adaptive functions became co‐evolutionarily entangled [1], each serving a non‐redundant role in immune defense and tissue homeostasis. Non‐redundancy between the two arms of vertebrate immunity is most dramatically demonstrated by the presence of primary immunodeficiencies that may involve innate functions, such as chronic granulomatous disease [2], or adaptive facilities, such as primary antibody deficiency [3]; other primary immunodeficiencies affect the hematopoietic system at the apex of leukocyte differentiation, such as reticular dysgenesis (aleukocytosis) [4]. However, whereas in the majority of vertebrates, innate and adaptive facilities co‐exist, an evolutionarily highly successful group of vertebrates, the deep‐sea anglerfishes, have abolished adaptive immunity altogether [5]; their survival most likely depends on restructured and augmented innate immune functions, illustrating the astounding plasticity of vertebrate immune systems [1].

## 2 Origin of Somatic Diversification

The evolutionary landscape that afforded a selective advantage to the early adaptive immune system remains a matter of speculation. One plausible hypothesis posits that it evolved to manage the ever‐increasing complexity of the microbiome that is associated with external and internal surfaces [6]; this view is supported by the presence of dysbiosis in primary immunodeficiencies [7, 8].

From an evolutionary point of view, the germline‐encoded components of innate immune facilities are, to a first approximation, identical between individuals of the same species. They are subject to Darwinian selection on evolutionary time scales to avoid physiologically relevant interaction with self structures and to preserve the recognition capacity for non‐self components. Indeed, mutations in innate components are often associated with severe autoinflammatory (sterile) pathology, such as that observed with malfunctions of the TNF signaling pathway [9]. By contrast, when it comes to somatically generated receptors, an additional and entirely different kind of quality control is required to ensure their functionality and self tolerance: operating at the level of each individual, self‐reactivity arising in the emerging repertoire is either eliminated by deletion of clones expressing undesirable receptor specificities [10] or by dedicated suppressive mechanisms [11]. Note, however, that Darwinian selection remains the major driving force also for such somatically generated receptors. First, it shaped the kind, number, and sequence composition of the genetic elements that are subject to the somatic diversification process, as exemplified by the presence of multiple isotypes of antigen receptors in jawless [12] and jawed [13] vertebrates. Second, it facilitated the domestication of genome modifiers for their immune‐related functions, as exemplified by the conversion of an ancient integrase into a recombinase that functions in V(D)J recombination [14]. Moreover, to tame the self‐destructive potential of the emerging somatic diversification processes, Darwinian selection favored the emergence of “dual‐use”‐type of lymphoid tissues, integrating lymphopoiesis and quality control, for instance in the thymus [15], and the emergence of dedicated regulatory cell types that function in the periphery [16]. It is, however, unclear which, if any, aspects of the molecular mechanisms that became an integral part of somatic quality control were re‐purposed from unrelated ancient facilities, or evolved de novo. As an example for the former scenario, the MHC peptide presentation system has been proposed to represent a repurposed sexual selection device [17].

## 3 Step‐Wise Elaboration of the Somatic Diversification Process

**FIGURE 1:**
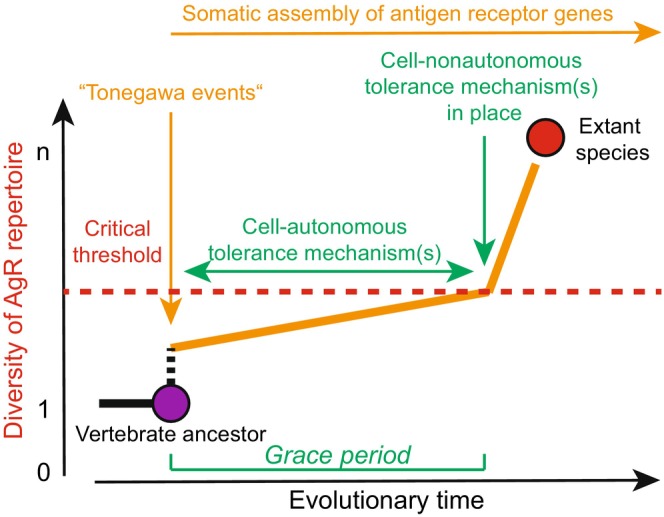
Evolutionary trajectory of somatic diversification and associated quality control mechanisms. The facility for somatic diversification of antigen receptors was initiated when genes encoding different types of cell surface molecules became disrupted in the distinct lineages emerging from the common vertebrate ancestor. Here, these foundational mutagenic incidents are referred to as “Tonegawa events,” named after the discoverer of somatic recombination of immunoglobulin genes [19]. It is thought that an ancestral, leucine‐rich repeat‐containing (LRR) platelet glycoprotein receptor‐like gene was disrupted in the lineage giving rise to jawless vertebrates [18], whereas an immunoglobulin domain‐encoding gene was mutagenized in the lineage giving rise to jawed vertebrates [20]. These singular founder loci gradually became more complex, particularly with respect to the genetic elements available for assembly, and eventually gave rise to gene families with similar properties: VLRs in jawless vertebrates, and IGs and TCRs in jawed vertebrates. In the initial stages of the implementation of the somatic diversification process, the number of genetic elements available for assembly of VLR‐type and Ig‐type of antigen receptors likely constrained the overall diversity of the resulting repertoires. Other cell‐intrinsic features, such as the microhomologies present at the ends of coding segments of Ig‐type receptors may have additionally limited excessive diversity [21], trading immunologically desirable receptor diversity against the risk of destructive self‐reactivity. Still, at some point in time, the diversity of reassembled sequences must have become so large that the maintenance of this unique self/nonself discrimination facility of the adaptive immune system necessitated the elaboration of dedicated cell‐nonautonomous quality control systems, such as the presentation of peripheral self‐antigens [15] in order to generate self‐tolerant repertoires; one such factor contributing to a sudden increase of diversity may have been the emergence and recruitment of terminal deoxynucleotidyl transferase (TdT) for non‐templated addition of nucleotides at the ends of double‐strand breaks [22, 23]. The duration of a hypothetical transitional (“grace”) period, during which additional safeguards evolved, is unknown. However, current evidence suggests that cell‐nonautonomous mechanisms may have emerged before the split of jawless and jawed vertebrates occurred [24, 25]; if true, this would imply that either one of the extant diversification systems preceded the other, or that early vertebrates relied on a diversification system that was lost during evolution. Likewise, it is not known whether the quality control processes referred to as central tolerance in primary lymphoid tissues emerged before those referred to as peripheral tolerance mechanisms that require distinct regulatory cell types.

When considering the early stages of the emerging somatic diversification process, it is reasonable to assume that the initial diversity of the somatically generated repertoire of antigen receptors was so low that it did not cause deleterious immunopathology, yet high enough to provide a selective advantage for certain physiological processes, including but not limited to immune defense. Both VLR‐type receptors of jawless vertebrates and the V(D)J‐type of receptors of jawed vertebrates are thought to have emerged after distinct mutagenic events that disrupted the contiguity of the ancestral precursors. For the VLR system, a platelet glycoprotein receptor‐like gene is considered to be the subject of the initial modification [18] (Figure 1). As a result, all descendants are incomplete in their germline configuration and require somatic assembly via a gene conversion‐like process utilizing leucine‐rich repeat‐domain encoding genomic cassettes to form complete genes [26]. The number and type of such cassettes determine the magnitude of the resulting repertoire of the extant VLR genes, with sequence constraints imposed by the gene conversion process.

The founding member of the V(D)J‐type receptors is thought to have emerged after the insertion of a transposon [19] into an exon encoding part of the extracellular domain of a cell surface receptor [20]. Excision of the transposon restored the coding region, albeit in some instances, leaving a small “scar” at the original insertion site as a result of the DNA repair process. As with VLRs, subsequent duplication of the disrupted exon, comprising an upstream “V”‐like sequence and a downstream “J”‐like sequence, eventually allowed for a greater degree of diversity in the resulting collection of restored receptor genes.

Up to a certain degree of receptor diversity (as a result of the number of gene segments and their sequence composition), cell‐autonomous quality control mechanisms likely sufficed to avoid excessive self‐reactivity (Figure 1). This afforded early vertebrates with a grace period during which more sophisticated somatic quality control mechanisms could have evolved. Hence, during the time when somatic diversity was not yet life‐threatening, primary lymphoid organs may have acquired the capacity for cell‐nonautonomous quality control. In this way, elaboration and assessment for self‐reactivity of the primary receptor repertoire became spatially associated (Figure 1). Hence, the potential for greater diversity in the repertoire was contingent on efficient and sophisticated quality control mechanisms, such as the exposure of peripheral self‐antigens to developing lymphocytes [15]. Note that it is not known whether “central” tolerance mechanisms (i.e., those that are spatially and temporally linked to the site of lymphocyte differentiation in primary lymphoid organs) emerged earlier than “peripheral” tolerance mechanisms via specialized regulatory/suppressor cell lineages. However, from today's perspective, the most parsimonious scenario suggests that central tolerance in primary lymphoid organs is the evolutionarily older mechanism, as no regulatory cell types have yet been described in jawless vertebrates.

Interestingly, with increasing complexity of antigen receptor repertoires late in vertebrate evolution, additional cell‐intrinsic quality control strategies emerged in the lymphoid system, one example being invariant “surrogate” chains that form heterodimers with somatically assembled receptor chains: The VpreB and Lambda5 light chain surrogates pair with immunoglobulin heavy chains to give rise to the preBCR [27], and the preTα chain pairs with TCRβ chain [28]. The purpose of such heterodimers is to “test” whether the assembled receptor chains are correctly folded and capable of reaching the plasma membrane, and to then generate a signal that licenses the next steps of lymphocyte differentiation. This clearly represents an evolutionary novelty, as no such formation of a developmentally intermediate receptor is known for the γδTCR. Given the astounding structural similarities between the immune systems of jawed and jawless vertebrates in terms of the sequence diversity of antigen receptor repertoires, it will be interesting to determine whether somatically assembled VLRs also require the (possibly only transient) association with an invariant VLR‐type (or other) molecule during the transition of an immature to a mature stage during lymphocyte differentiation.

In sum, quality control mechanisms serve two purposes. First, they tame the self‐destructive potential of somatically assembled receptor repertoires. Second, it emerges that many structural and mechanistic aspects of receptor repertoire formation are under Darwinian selection. They establish several checkpoints during the differentiation process of lymphocytes to limit the metabolic investments incurred by the production of non‐functional or even harmful components of repertoires, avoiding the formation of nonfunctional and/or dysfunctional effector cells and potential autoimmune reactions.

In this review, we discuss recent findings that illuminate some of the phylogenetic signals that accompany the evolution of somatic diversification process of antigen receptors. We briefly touch upon the characteristics of the VLR antigen receptor assembly process in jawless vertebrates, but dedicate the major part of the review to a discussion of the TCR system of jawed vertebrates and will make reference to the BCR assembly only for comparative purposes. We highlight the evolution of the antigen receptor genes and explore how their germline‐encoded components and the mechanisms of their assembly process determine the diversity of primary repertoires. Hence, we seek to provide initial answers to the following questions: How is diversity regulated at the individual and species level? Are there unifying factors in immune repertoire diversification across species?

## 4 A Systems Level View of Adaptive Immunity

The process of somatic diversification of antigen receptors is shaped, but not entirely determined, by Darwinian selection; hence, somatic quality control mechanisms operating during ontogeny have evolved to avoid, or at least mitigate, the risk associated with inadvertent self‐reactivity. Whereas repertoire structure is clearly not entirely predictable, recent work has begun to question assumptions of an essentially unlimited diversity in antigen receptor repertoires. Thus, even though the anticipatory mode of antigen receptor diversity represents the cornerstone of adaptive immunity and is considered to be its defining feature, natural selection may not necessarily favor maximal diversity of immune repertoires. We suggest that comparative studies of immune repertoires across all clades of jawed vertebrates promise to identify the evolutionarily malleable components of the somatic diversification process, eventually resulting in more realistic estimates of receptor diversity. A meaningful interpretation of receptor diversity must be considered against factors that operate at an organismic level, such as habitat structure, pathogen exposure, population size, and life history (life span, body size, reproductive strategy, etc.). These factors likely determine the extent to which adaptive facilities are required for immune defense and tissue homeostasis. Hence, they directly impact the evolutionary trends that shape the molecular components of the somatic assembly process and post‐assembly quality control mechanisms. In Table 1, we summarize several of these evolutionarily plastic aspects, some of which were addressed by the comparative studies discussed below.

**TABLE 1:** Evolutionarily plastic aspects of repertoire formation.^a^

Combinatorial diversity	Junctional diversity	Post‐assembly modifications
AgR isotypes	*Microhomology*	Exposure to peripheral self‐antigens in primary lymphoid organs
Number and types of elements available for assembly	*RSS position*	*Clonal deletion*
Chromosomal arrangement of elements	*Exonuclease activity*	*Lineage diversion for regulatory purposes*
*RSS quality*	*TdT activity*	*AgR revision*
		*Somatic hypermutation*
		*Class switch recombination*

^a^ Parameters that—based on current knowledge—are unique to the Ig‐based AgRs of jawed vertebrates are indicated in italics; other aspects are common also to the VLR‐based system of jawless vertebrates.

## 5 The Assembly Process of VLR Antigen Receptors in Jawless Vertebrates

At present, six VLR isotypes are known, five of which are attributed to distinct T cell lineages, whereas one isotype, VLRB, is uniquely expressed in B‐like lymphocytes (for the description of the most recent addition to the T‐like lineages, see [29]). All VLR receptors [12, 30] are composed of leucine‐rich repeat (LRR) modules and membrane‐bound (in the case of VLRB via GPI linkage) of conserved structure, comprising an N‐terminal cap (LRRNT), a short LRR module (LRR1), variable numbers of 24‐amino acid long LRRs (LRRVs), a connecting peptide of 12 amino acids, a C‐terminal cap (LRRCT), and an invariant stalk region. In the germline configuration, all VLR genes are incomplete, encoding only parts of the N‐ and C‐terminal segments. The individual elements required to generate a complete gene are found in the vicinity of the incomplete germline genes [12]. In developing lymphocytes, the activity of cytidine deaminases is required [18, 31] to support a gene conversion‐like process that copies missing elements into the germline gene [26].

VLR assembly is a combinatorial process [12]. Neither junctional diversity nor post‐assembly modifications have yet been described, indicating that the diversity of the repertoire of mature VLRs is entirely dependent on the type and number of elements that are joined together. As these elements are all encoded in the germline, it is possible—at least in principle—to know the outcome of the assembly process, and hence the specificity of receptor‐ligand interactions. The gene conversion process is constrained by the complementary nature of the relevant sequences, which dictates the sequence of assembly events; given the large number of LRRVs in the germline [12, 32], in addition to a certain degree of cassette sharing between the VLRs expressed in T‐like cells [29, 33, 34], the diversity of VLR sequences is thought to be very large [12]. However, because no exhaustive repertoire studies have yet been performed for all VLR isotypes (with the exception of VLRB [35]), the actual diversity of VLR repertoires and their clonal structures remains unclear. Of note, individual lamprey and hagfish species are very different in size, likely impacting the fraction of the possible combinatorial diversity that is present in the repertoires. So‐called brook lampreys, such as *Lampetra planeri* , measure about 10 cm in length, whereas the sea lamprey *Petromyzon marinus* can reach up to 120 cm; likewise, small hagfish species, such as *Myxine kuoi, also* measure around 10–12 cm, whereas the largest known species, *Eptatretus goliath* rivals the size of the sea lamprey. Accordingly, body weights vary by two orders of magnitude, from about 10 g to 2 kg. Comparative analyses of the numbers and types of the germline cassettes of VLRs in small and large species have not yet been reported, although it would be interesting to know whether the body size (and by inference the number of lymphocytes) scales with the germline repertoire of VLR gene elements. With respect to the overall structure of VLR isotypes, a peculiar difference exists in the size distributions of VLRs belonging to the T‐like lineages (VLRA, VLRC. VLRD, VLRE, VLRF), and the B‐lineage restricted VLRB isotype. Whereas the former are characterized by distributions centered around 4 LRRV elements [32, 36], VLRB assemblies exhibit a Poisson distribution of the number of LRRV elements [35, 37]. The size distribution in peripheral T‐like VLRs is attributed to an as yet unknown selection process acting upon the pre‐selection repertoire [36], most likely occurring in the thymus equivalent of lampreys [38]. It has been proposed that the formation of the peripheral repertoire may additionally involve a selection process akin to central tolerance formation in jawed vertebrates [15, 24, 25], but the underlying mechanism remains elusive. Nonetheless, given that the combinatorial diversification process results in predictable specificity, it is not unreasonable to assume that cell‐nonautonomous quality control measures required to curtail potential self‐reactivity in VLR repertoires, if present, are less demanding than those required for the antigen receptor repertoires of jawed vertebrates.

## 6 The Assembly Process of Antigen Receptors in Jawed Vertebrates

As with VLRs, the germline configurations of TCR [39, 40] and BCR [41] genes are incomplete and require a somatic assembly process [19, 42, 43] by the RAG recombinase [44, 45] to form functional units. The generation of antigen receptor repertoires via combinatorial V(D)J recombination is far from random [46, 47]. It is influenced by the accessibility, location, and orientation of variable (V), diversity (D), and joining (J) elements relative to the RAG recombination center [48]. Of note, selective sweeps affecting the number and sequence diversity of variable region genes of immunoglobulins and T cell receptors occur even among different populations within one species [49, 50, 51]; moreover, species‐specific sequence variations among recombination signal sequences flanking the V, D, and J elements, in the conserved heptamer and nonamer sequences, the associated spacer sequences, and in flanking coding sequences also determine the final outcome of recombination [47, 52, 53, 54, 55, 56]. All these parameters (ranging from control of chromatin accessibility to the precise sequences of recombination signal sequences) are evolutionarily selected and impact the type of RAG‐mediated recombination mechanism, that is, directional scanning or diffusional joining, as exemplified by the different modes of Ig heavy chain and IgK light chain assemblies [57, 58].

Importantly, however, in contrast to VLRs, the sequence diversity of antigen receptors is governed not only by combinatorial mechanisms but largely determined by junctional diversity. Junctional diversity is generated via both losses and additions of nucleotides after the double‐stranded break induced by the RAG recombinase. Whereas the signal joints are precise fusions, coding DNA ends are subject to exonucleolytic degradation [59, 60], which removes germline‐encoded nucleotides. In addition, coding joints may be modified by additions of nucleotides that can either be template‐dependent [61, 62] (termed P nucleotides) or non‐templated [59, 63, 64] (termed N nucleotides), although the latter is associated with certain nucleotide preferences [65]. As a result, sequences encompassing the junctions—commonly referred to as complementarity determining regions (CDR3)—are, to a certain degree, unpredictable. On the other hand, diversity is constrained by short stretches of sequence identities (micro‐homologies) that are present at the coding ends of the elements that are joined during the V(D)J recombination process of TCRs [66, 67, 68, 69] and BCRs [70, 71]; this phenomenon is particularly noticeable when terminal deoxynucleotidyl transferase (TdT) is not expressed during certain stages of lymphocyte development [72], or after genetic ablation of TdT [63, 64]. As the junctional sequences form part of ligand binding surfaces, it follows that the specificity of the receptors is not entirely predictable from the germline sequences. This has obvious consequences for the design of quality control mechanisms to mitigate undesirable self‐reactivity; they are thus likely more complex than those required for the VLR system in jawless vertebrates. In summary, although a number of evolutionarily selected parameters impinge on the extent of CDR3 diversity, the ontogenetic (and hence unpredictable) part of the CDR3 region is substantial enough to warrant the elaboration of dedicated cell‐nonautonomous somatic quality control measures (Figure 1).

## 7 Challenges Associated With Experimental Analysis of Antigen Receptor Diversity

To date, a comprehensive analysis of antigen receptor repertoires has not been achieved for any species. Biological aspects and technical issues conspire to allow one to take only a snapshot of antigen receptor diversity. For instance, the largely divergent body sizes constitute a major problem. There is a body mass difference of more than 11 orders of magnitude between the smallest known vertebrate (minifish, *Paedocypris* spp., about 10 mg) and the largest (blue whale, *Balaenoptera musculus* , about 200,000 kg); even for the mouse (body mass of about 20 g), it has not yet been possible to arrive at a complete representation of antigen receptor diversity. Any attempt at a representative analysis of repertoires is further complicated by the known differences in tissue distribution of lymphocyte types. Hence, the antigen receptor repertoires of different species are often compared on the basis of material obtained from different body compartments, for instance spleen, peripheral blood, under the assumption that the physiology of immune organs is comparable. This clearly is not the case, especially when one compares repertoires across different clades of vertebrates, such as fish and mammals, which differ in the presence and/or histological organization of lymphoid tissues. Another aspect concerns the different assembly modes across different antigen receptor genes. This problem is illustrated by the finding that the activity of TdT is regulated not only during development, but also in a species‐specific manner. For instance, assembled TCRα chain genes of teleosts have little junctional diversity, whereas this is a prominent feature of mammalian TCRα gene assemblies [21] (see below).

Additional biological parameters, such as developmental maturity, immune activity, etc., and technical issues also affect the experimental determination of diversity. Whereas information on the nature of antigen receptors can easily be obtained for cells in suspension (i.e., blood), it becomes a challenge with tissue‐resident cells, owing to the difficulty of disintegrating the tissue material without destroying, perhaps even to different degrees, the relevant cells. Even then, faithful representation of antigen receptor mRNAs in sequencing libraries remains difficult to achieve, especially given the fact that different lymphocyte types contain different quantities of mRNAs (for instance, pre B cells vs. plasma cells). To determine the exact composition of the CDR3 region of immunoglobulin and TCR genes, only relatively short stretches of sequences suffice, making repertoire studies amenable to established high‐throughput short read technologies; by contrast, analyses focusing on the extent of somatic hypermutation of these genes require longer sequence reads, raising the complexity and cost of the experiments. This problem is magnified in analyses of VLR repertoire. Here, the diversity of long tandem LRR arrays can only be assessed when the complete molecule is sequenced, requiring read lengths of up to 1.5 kb [35].

## 8 Quantification of Antigen Receptor Diversity

In an ideal situation, one would be able to comprehensively determine the entire repertoire of an organism. A comparison to the respective germline sequences then provides information on the usage of individual elements, potential biases in the pairing pattern of heterodimeric chains, and reveals the extent of post‐assembly modifications, such as receptor editing and somatic hypermutation (SHM); in addition, the clonal structure that is, the size of clones and their genealogical relationships, would be revealed.

Shortly after the general principles of diversity generation were established, and the genomic structures of BCR and TCR antigen receptor genes were revealed, attempts were made to estimate the theoretical diversity of the antigen receptor repertoires. These calculations were based on the multiplication of the number of genetic elements by the number of possible junctional sequences (as a result of removal of germline encoded nucleotides and/or addition of non‐templated nucleotides) [73]. The results suggested that the number of potential sequences thus estimated would exceed the number of lymphocytes by several orders of magnitude. If so, this would have the important implication that even within one species, repertoires of individuals are essentially private, and that the number of shared clonotypes individuals would be negligible, even for genetically identical twins. It thus came as a surprise when high throughput measurements of human CD8+ T cell receptor repertoires indicated that the overlap of CDR3 sequence repertoires in the naïve CD8+ populations of any two individuals is approximately 7000‐fold larger than predicted and that it appeared to be independent of the degree of human leukocyte antigen matching [74]. These findings suggested that biases in the V(D)J recombination process leave a recognizable imprint in the post‐selection repertoire. Additional evidence for a hardwired bias also came from studies, in transgenic mouse models, that revealed species‐specific differences in the clonal diversity of TCRα and TCRβ sequences selected on either mouse or human MHCII alleles [75]; remarkably, this study also revealed statistically different properties of the CDR3 regions of mouse and humans, mirroring findings from direct comparative analyses [76]. Nonetheless, given that comprehensive measurements of total receptor diversity are currently beyond reach, other strategies to explore repertoire diversity were developed.

The use of information theory is one such promising approach [77]. In the present context, entropy is a measure of how unpredictable a particular receptor sequence is; in other words, a measure of how surprising it is to find a particular sequence in the repertoire. In information theory, entropy corresponds to the average information content in a random variable; it is measured in bits and can be used, according to the original intent, to determine the limits of data compression and the efficiency of communication over the noise of the communication channel, essentially distinguishing noise from the meaning of a message. Hence, when applied in the present context, if the entropy of a particular antigen receptor repertoire is higher than another, then the system of the former is capable of generating higher diversity.

A key advantage of using entropy to estimate the diversity of repertoires is that it is weighted by the actual repertoire usage, unlike the original calculations that could not (for lack of relevant information) consider the biases introduced by recombination frequency and CDR3 structure. If we assign an entropy to each set of sequences of every possible CDR3 length, the final entropy of the immune repertoire will be the weighted average of their respective entropies, plus a small contribution from the entropy of their distribution. Additionally, we can use mutual information, a quantity that expresses how much entropy from one variable is encapsulated in another variable, to estimate how much additional randomness (technically “conditional entropy”) is present in the CDR3 region after knowing the V and J segments, thus quantifying the relative contributions of junctional and combinatorial diversity with the same metric. This is especially useful when comparing the repertoire of different species that, as we will see, sometimes vary in their strategies for V(D)J recombination.

## 9 Antigen Receptor Repertoires of the Smallest Vertebrate

The maintenance of an adaptive immune system invariably incurs a metabolic cost, owing to the necessity of producing and maintaining the signature cell and tissue types. Activation of immunity triggers a complex physiological response that, in mice, results in hypometabolism and hypothermia, indicating that immune defense is costly [78], but may be well worth the benefit gained [79]. We have approached this problem from a different perspective. We reasoned that body miniaturization might be associated with a reduction in adaptive (and possibly also in innate) immune facilities. We focused on the nature and diversity of antigen receptor repertoires [80] in the smallest known vertebrate, the minifish *Paedocypris* spp. [81]. To our surprise, we found that this small teleost possesses the canonical set of antigen receptors, the four TCR genes as well as Ig heavy and light chain genes. The unique biology of this species allowed us to carry out a near‐complete assessment of the different gene repertoires, as the fish harbors only around 50,000 lymphocytes. We then set out to compare the germline genes of the minifish with those of the zebrafish *D. rerio* that also belongs to the Cyprinidae clade of teleosts.

We found that the minifish possesses far fewer V and J gene elements in the TCRα, TCRβ, and IgH loci than zebrafish. Moreover, the number of overlapping sequences across individuals, commonly referred to as public clones, was much higher than expected. Thus, in this species, many of the lymphocytes express the same clonotypes, a highly unusual feature among the vertebrates (mostly mammals) that had been analyzed at that time. These results suggested that, given the limited capacity to express somatically assembled antigen receptors, individual antigen receptors exhibit a high degree of promiscuity in order to accommodate the structural diversity of potential ligands. In this context, it will be very interesting to examine the extent of deletion and/or pruning of the primary repertoires, and how the magnified risk of self‐reactivity associated with cross‐reactive receptors is dealt with.

## 10 Evolutionary Footprints in the V(D)J Recombination Process

**FIGURE 2:**
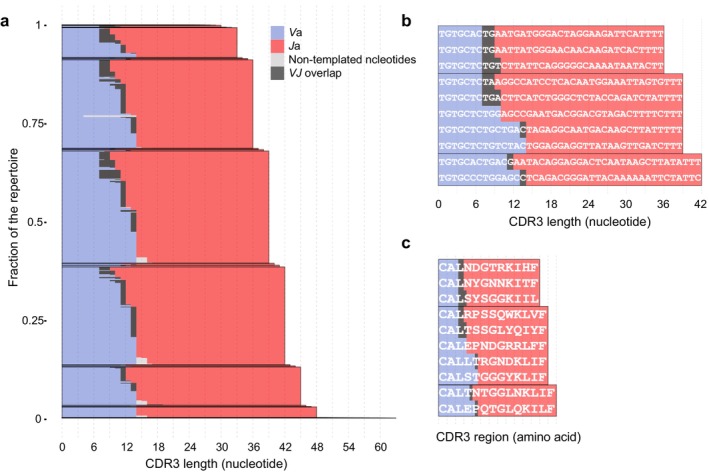
Contribution of microhomologies to the CDR3 region of *tra* assemblies of the zebrafish *D. rerio* . (a) The origins of nucleotide (nt) residues are indicated; variable genes (blue), joining genes (red), ambiguous origin (either V or J) (black) and non‐templated (gray). The height is proportional to the count of cDNA molecules. The sequences from three animals are combined. (b) Examples of CDR3 nucleotide sequences; color code is as in (a). (c) Conceptual translation of nucleotide sequences in (b); color code is as in (a).

**FIGURE 3:**
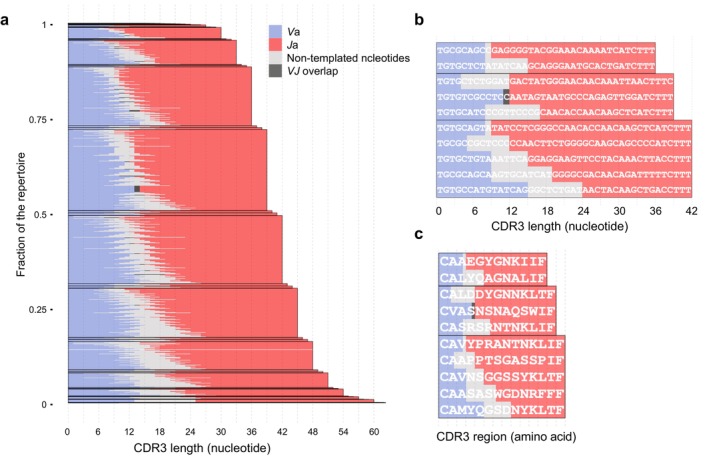
Contribution of microhomologies to the CDR3 region of *TRA* assemblies of the elephant *L. africana* . (a) The origins of nucleotide (nt) residues are indicated; variable genes (blue), joining genes (red), ambiguous origin (either V or J) (black) and non‐templated (gray). The height is proportional to the count of cDNA molecules. The sequences from three animals are combined. (b) Examples of CDR3 nucleotide sequences; color code is as in (a). (c) Conceptual translation of nucleotide sequences in (b); color code is as in (a).

**FIGURE 4:**
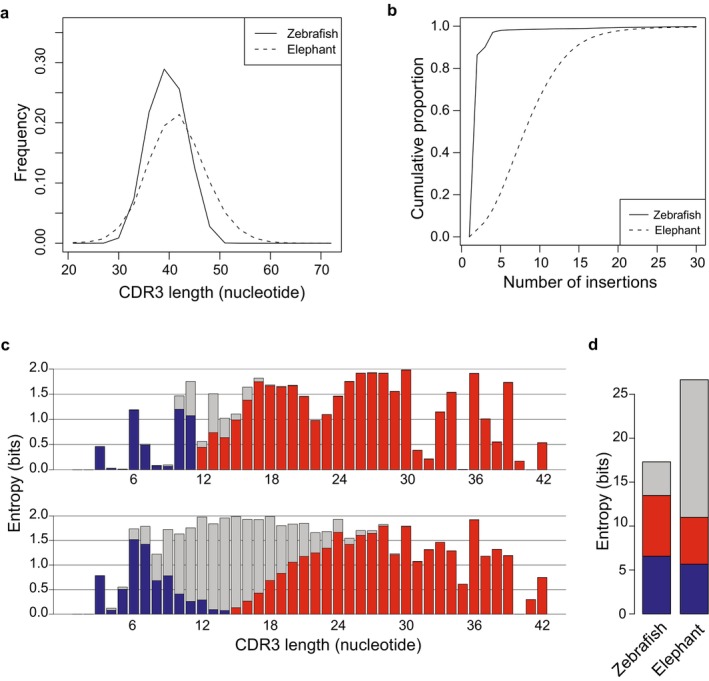
Characterization of *TRA* CDR3 regions. (a) CDR3 length distribution for cDNAs of zebrafish and elephant; the sequences from three animals are combined. (b) Cumulative distribution of CDR3 sequences as a function of nucleotide insertions. (c) Diversity at individual nucleotide positions for 42 nucleotide‐long CDR3 sequences. Indicated are germline‐dependent contributions to mutual information between nucleotide and gene (*V* gene, blue bars; *J* gene, red bars) and germline‐independent contributions (conditional entropy when *V* or *J* genes are known, gray bars). (d) Sources of total entropy; color code as in (c). Entropy values are derived from amino acid sequences.

The finding of unusually high proportions of public clonotypes in minifish antigen receptor repertoires hinted at the possibility that the generative rules for antigen receptor assembly might differ across species. We therefore embarked on a comparative study to include a wide range of vertebrates, from cartilaginous fishes to large mammals. For the present discussion, we will initially focus our attention to two representative species, the zebrafish *Danio rerio* and the African bush elephant *Loxodonta africanus*. The sequences of the CDR3 regions of TCRa assemblies presented in Figures 2 and 3, highlight the key difference in the extent of junctional diversity in these two species. Zebrafish assemblies represent the presumed ancient assembly type [21], and are marked by a large degree of microhomology‐directed recombination. Microhomologies are short identical sequences (typically ranging from 1 to 4 nucleotides) that are present at the ends of broken DNA strands; in the present case, this refers to the germline‐encoded nucleotides at the 3′‐ends of V regions, and the 5′‐ends of J regions (for antigen receptor genes with a V‐J configuration). These matching sequences bias the outcome of the V(D)J recombination process thereby reducing junctional diversity; as a result, the sequence of such junctions exhibit nucleotides whose origin, from V or J segments, cannot be ascertained. This phenomenon is apparent from the frequent overlap of nucleotides at the ends of V and J regions (Figure 2a). Accordingly, the degree of junctional diversity is low (Figure 2b), and translates into CDR3 sequences that are largely predictable from V and J germline sequences (Figure 2c). By contrast, elephant assemblies exhibit a considerable degree of junctional diversity, and V‐J sequence overlaps are rare (Figure 3a,b); as a result, the CDR3 regions thus contain up to three non‐templated amino acids (Figure 3c). This difference is reflected in typically longer CDR3 regions in elephant assemblies (Figure 4a). In zebrafish, the cumulative distribution of insertions (i.e., non‐templated nucleotides) indicates that about 98% of TCRα junctions exhibit 3 or fewer insertions, whereas up to 19 nucleotides are required to describe 98% of elephant assemblies (Figure 4b). To illustrate this dramatic difference, we present the diversity at individual nucleotide positions for 42 nucleotide‐long TCRα sequences for both zebrafish and elephant, demonstrating that germline contributions to mutual information between nucleotide and V and J genes dominates the overall entropy in zebrafish (Figure 4c). The total entropy in elephant TCRα assemblies is almost twice as high as in their zebrafish counterparts (about 27 bits versus about 17 bits), the main difference being afforded by germline‐independent contributions; junctional sequences contribute 4 bits for zebrafish and to about 16 bits for elephant (Figure 4d).

**FIGURE 5:**
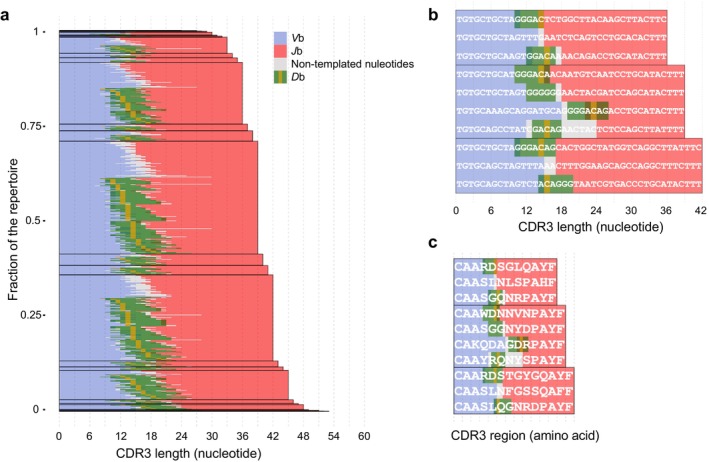
Contribution of microhomologies to the CDR3 region of *trb* assemblies of the zebrafish *D. rerio* . (a) The origins of nucleotide (nt) residues are indicated; variable (*V*) genes (blue), joining (*J*) genes (red), diversity (*D*) genes (green/yellow), and non‐templated (gray). The height is proportional to the count of cDNA molecules. The sequences from three animals are combined. (b) Examples of CDR3 nucleotide sequences; color code is as in (a). (c) Conceptual translation of nucleotide sequences in (b); color code is as in (a).

**FIGURE 6:**
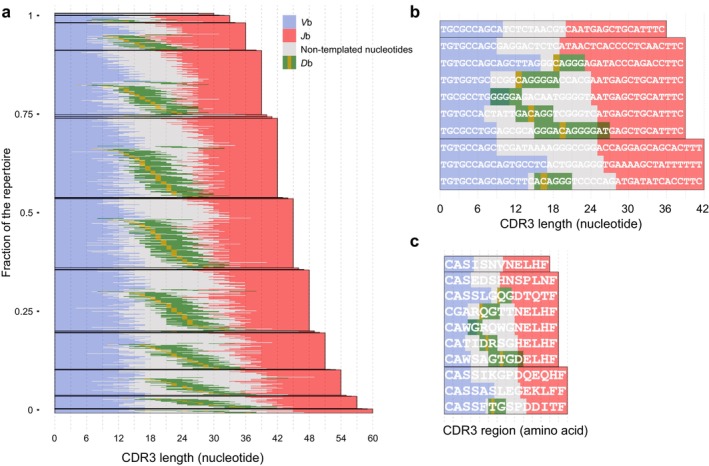
Contribution of microhomologies to the CDR3 region of *TRB* assemblies of the elephant *L. africana* . (a) The origins of nucleotide (nt) residues are indicated; variable (*V*) genes (blue), joining (*J*) genes (red), diversity (*D*) genes (green/yellow), and non‐templated (gray). The height is proportional to the count of cDNA molecules. The sequences from three animals are combined. (b) Examples of CDR3 nucleotide sequences; color code is as in (a). (c) Conceptual translation of nucleotide sequences in (b); color code is as in (a).

**FIGURE 7:**
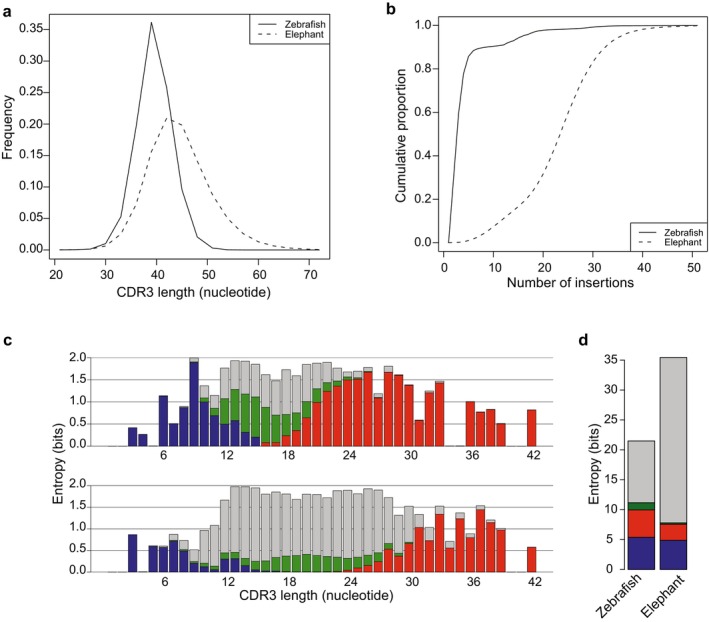
Characterization of *TRB* CDR3 regions. (a) CDR3 length distribution for cDNAs of zebrafish and elephant; the sequences from three animals are combined. (b) Cumulative distribution of CDR3 sequences as a function of nucleotide insertions. (c) Diversity at individual nucleotide positions for 42 nucleotide‐long CDR3 sequences. Indicated are germline‐dependent contributions to mutual information between nucleotide and gene (*V* gene, blue bars; *J* gene, red bars; *D* gene, green bars) and germline‐independent contributions (conditional entropy when *V* or *J* genes are known, gray bars). (d) Sources of total entropy; color code as in (c). Entropy values are derived from amino acid sequences.

This species‐specific difference generally holds also for TCRβ assemblies (Figures 5 and 6). Of note, in zebrafish TCRβ assemblies, sequences of D elements are almost always recognizable and are sandwiched between V and J elements that essentially lack N‐regions (Figure 5a). As a result, the contribution of non‐templated sequences to CDR3 regions is minimal (Figure 5b,c). The structure of TCRβ assemblies in the elephant repertoire is dramatically different. Although the contributions of D sequences to CDR3 diversity are still obvious (Figure 6a), the extended regions of non‐templated sequences (Figure 6a,b) result in binding surfaces exhibiting a high proportion of non‐templated amino acid sequences (Figure 6c). Correspondingly, CDR3 regions in elephant are much longer (Figure 7a,b). With respect to the contributions of non‐templated sequence to the overall entropy, about half of the total entropy of 22 bits is contributed by germline‐independent contributions of V, D, and J elements in zebrafish, whereas this amounts to about 80% of the 36 bits in elephant TCRβ assemblies (Figure 7d).

These data indicate that the combined effects of exonucleolytic loss of nucleotides [60, 82, 83] and TdT‐mediated additions [63, 64] are only minor contributors to junctional diversity in evolutionarily more ancient species (here exemplified by zebrafish), but assume a dominant role in mammals. Interestingly, the activity of the exonuclease(s) responsible for the loss of nucleotides at the ends of coding segments during the V(D)J recombination is subject to modulation via the NFΚB pathway [83]; likewise, the activity of TdT is known to be developmentally regulated in T and B cell differentiation [84, 85]. These properties provide a mechanistic basis by which junctional diversity can be subjected to evolutionary selection.

The low entropy values obtained for antigen receptor repertoires of teleosts can be explained by the presence of microhomologies at the end of coding regions, the regular positions of associated RSS segments, and apparent low activity of TdT. This appears to be a characteristic of the teleost clade, as it is independent of body mass, in our experiments spanning 0.2 g (minifish) to 1500 g (rainbow trout) [21]. In birds and mammals, this pattern becomes less obvious, most likely due to the erosion of microhomology and the altered regulation of TdT activity. The constrained CDR3 diversity in TCRα assemblies of teleosts allows for only three possible lengths for each V‐J pairing. In some species, this apparent limitation can be offset by the presence of a large number of J segments that are present in teleost fishes; for example, zebrafish that exhibit an extremely limited TCRα repertoire possess almost twice as many Jα gene segments as humans. As a result of this compensatory adaptation to stereotypical recombination, the overall CDR3 length distribution of zebrafish and the other teleost species we studied exhibits the familiar Gaussian distribution of lengths seen in mammals. It will be interesting to see if a reduction of J elements in the TCRα locus of species with comparable body mass can be compensated for by the “violation” of the stereotypic teleost recombination type by, for instance, increasing junctional diversity via greater TdT activity or degradation of the germ‐line encoded microhomology at coding ends.

Microhomologies that are often found at ends of coding regions are considered the remnants of the target‐site duplications that are associated with the foundational transposon insertion event initiating the V(D)J recombination mechanism. They are known to exert a dominant influence on the outcome of recombination, but only if enzymatic modification of coding ends is kept at bay, as was first described for a subset of fetal antigen receptors in mammals [64, 86]. From an evolutionary point of view, TdT (and perhaps also exonuclease) activities appear to have been recruited to the diversification process at more advanced stages of adaptive immunity, when quality control mechanisms allowed for greater levels of unpredictability in the spectrum of antigen binding surfaces.

## 11 Antigen Receptor Repertoires in Context

**FIGURE 8:**
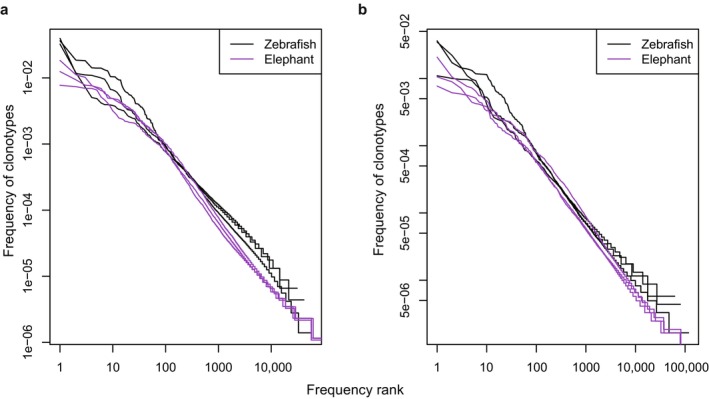
Clonal distributions of TCR chains from zebrafish and elephant. (a) The proportion of *TRA* clonotypes is plotted as a function of their frequency rank in the repertoire. (b) The proportion of *TRB* clonotypes is plotted as a function of their frequency rank in the repertoire. The results of these animals are shown. The numbers of represented cDNA molecules by specimen were as follows. *TRA*: 718,479; 227,750; 151,878 for zebrafish, and 870,408; 922,718, 861,823 for elephant. *TRB*: 606,268; 228,259; 172,881 for zebrafish, and 1,077,680; 1,219,169; 1,215,116 for elephant. A small number of clonotypes constitute the bulk of cDNA molecules, whereas most clonotypes are represented less frequently in the repertoires. For both assemblies, there is a trend for fewer large clonotypes in the elephant. To demonstrate this effect, downsampling was not performed.

As discussed above, the body mass of vertebrates vastly differs, spanning more than 10 orders of magnitude. It is likely that body size also scales with the number of lymphocytes; one important caveat here is that the structure of lymphoid tissues greatly differs between mammals and other vertebrates, particularly with respect to the presence and organization of secondary lymphoid tissues, such as spleen and lymph nodes. Despite these anatomical differences, the frequency‐rank distributions of antigen receptor clones in minifish follow a power law [80], irrespective of receptor isotype; this has previously been described for mammalian repertoires as well [87, 88, 89, 90, 91]. Self‐similarity is frequently found in natural systems [92], and is mathematically described by the fractal dimension, which, in the present context, is given by the proportion of the logarithm of clone size (determined as the frequency of a clone) by logarithm of rank [93]. To illustrate the remarkable self‐similarity of antigen receptor repertoires, we compared the TCRα and TCRβ assemblies for three individuals each of zebrafish and elephant (Figure 8). Strikingly, the slopes of the curves (equivalent to the fractal dimensions of the repertoires) for both TCRα and TCRβ assemblies are very similar for both species, despite the fact that their body masses differ by a factor of ~10^7^. An analysis of lamprey VLRB repertoires also suggests that the clonal structure follows a power law [35]. These observations indicate that the power law describing the clonal distribution pattern is a general property of somatically assembled antigen receptor repertoires across vertebrates of different size and evolutionary distance.

**FIGURE 9:**
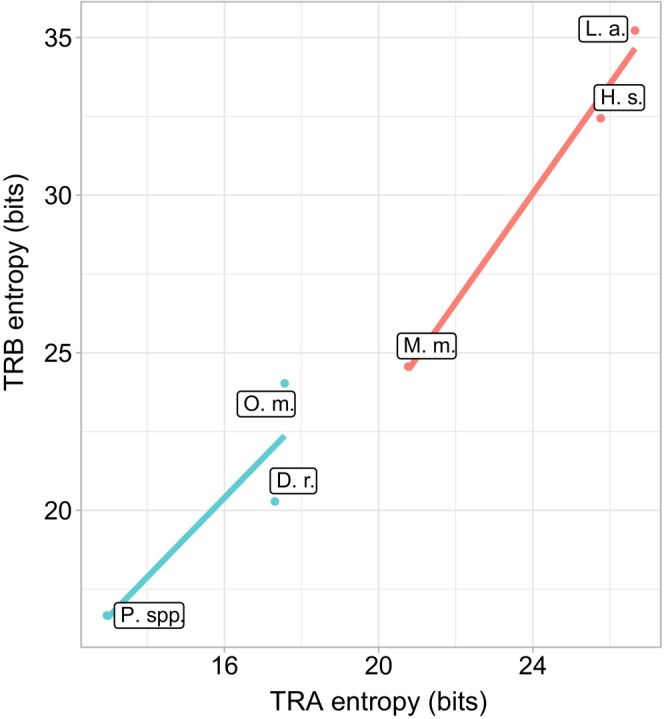
Linear relationship between *TRA* and *TRB* entropies in various species. P. spp., *Paedocypris* spp. (minifish); D. r., *Danio rerio* (zebrafish); O. m., *Oncorhynchus mykiss* (rainbow trout); M. m., *Mus musculus* (mouse); H. s., *Homo sapiens* (human); L. a., *Loxodonta africana* (elephant). Data points for teleosts are in blue color, those for mammals are in red color. Entropy values are derived from amino acid sequences.

Another curious phenomenon becomes apparent when one compares the overall entropy values for TCRα and TCRβ assemblies in individual species. Irrespective of the particular recombination strategy used, considering the presence or absence of microhomologies and the difference in TdT activity, the ratio of entropies is relatively invariant, with TCRβ always making a greater contribution (Figure 9). For instance, in the bamboo shark, the ratio is 0.76; it amounts to 0.70 for minifish and to 0.73 for rainbow trout; for the elephant it is recorded at 0.76. The substantial degree of TCRα publicity prompts us to propose that the TCRα chain in the TCRαβ heterodimer might function akin to a pattern‐recognition receptor, interacting somewhat promiscuously with a particular class of antigens, whereas the corresponding TCRβ supports a more fine‐grained differentiation of ligands. Evolutionary remnants of this type of division of labour might have survived in the form of the known T cells that express semi‐invariant heterodimers, such as iNKT and MAIT cells [94, 95]. Thus, what might have been a predominant feature of the TCRαβ heterodimer at early stages of vertebrate evolution, may have become relegated to a small subset of the much expanded repertoire at later stages. In this context, it will be interesting to determine whether non‐mammalian species also possess such semi‐invariant receptor signatures, associated with particular lymphocyte lineages, that in extant species are outnumbered by the “classical” variable receptor combinations.

**FIGURE 10:**
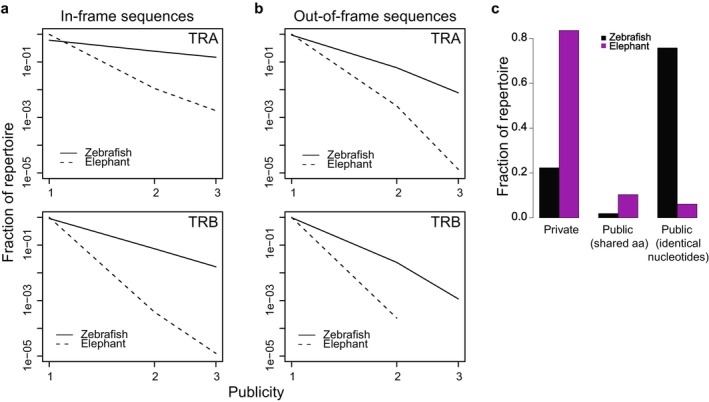
Publicity of *TRA* and *TRB* clonotypes in zebrafish and elephant. (a) In‐frame CDR3 nucleotide sequences; *TRA*, top panel; *TRB*, bottom panel. (b) Out‐of‐frame CDR3 nucleotide sequences; *TRA*, top panel; *TRB*, bottom panel. The *x*‐axis denotes the degree of publicity: 1; sequences that occur in only one individual; 2, sequences that occur in two individuals; 3, sequences that occur in three individuals. (c) Fraction of the total repertoire of TRA assemblies that are unique among three individuals per species (Private; left columns), or share the amino acid sequence but not the nucleotide sequence (i.e., originating from independent assembly events and yielding the same amino acid sequences; middle columns), or share the exact nucleotide sequence (representing either descendants of independent assemblies with identical outcome, or expanded clones; right columns). For this analysis, 1,659,351 unique molecular identifiers of zebrafish (*n* = 6), and 2654,949 unique molecular identifiers of elephant (*n* = 3) were used without subsampling; this was done to highlight the fact that in zebrafish public TRA assemblies almost always share the same nucleotide sequence, reflecting the strong impact of microhomologies at the ends of V and J elements.

Our studies in minifish indicated that public clones are strongly connected to the unique (“private”) fraction of the repertoire and that this centrality is key to maintaining the connectivity in the network of individual clones [80]. The degree of publicity in TCRα and TCRβ repertoires is far greater in teleosts than it is in mammals, as illustrated in Figure 10a for zebrafish and elephant. The significant impact of the recombination mode on publicity is obvious from the analysis of out‐of‐frame clonotypes that essentially reflect the generative properties of the recombination process; here, the same difference as for the in‐frame clonotypes is observed between zebrafish and elephant (Figure 10b). For TCRα sequences, the major effect of the sequence‐driven recombination process can also be demonstrated by the fact that shared amino acid sequences in zebrafish are almost completely encoded in the nucleotide sequence, whereas this is not the case in the elephant TCRα assemblies (Figure 10c). Once reliable methods become available for in silico prediction of ligand specificity of TCRαβ, it will be interesting to examine whether a species‐specific signal can be detected in the fraction of the repertoire that is occupied by expanded clones, or whether specificities in the public repertoire rather reflect adaptations to a particular habitat. For humans, it may be instructive to examine public repertoires with respect to the known co‐diversification of the microbiome with human populations [96].

**FIGURE 11:**
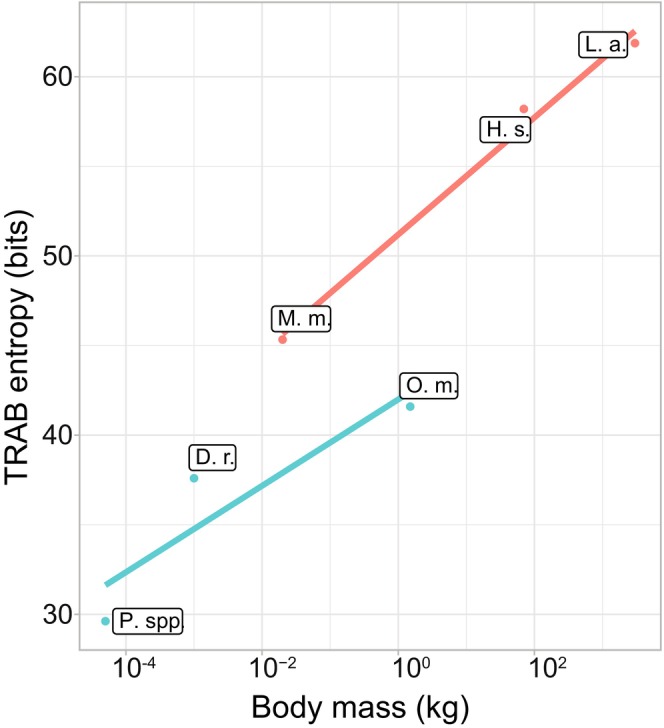
Relationship of total entropy of *TRAB* heterodimers to body mass. Note that the slopes of the regression lines are off‐set by several bits, indicating that mammals generally exhibit higher entropy. Entropy values are derived from amino acid sequences. A double‐logarithmic representation was used, as bits are mathematically linked to logarithm (to base 2); the *x*‐axis is also scaled to logarithm (to base 10). For this analysis, no subsampling was used for the following reason: subsampling would increase mutual information and hence reduce the entropy estimates, impacting a fair representation of the repertoires, particularly for the repertoires of the minifish and the elephant, the two species situated at the extreme ends of the mass spectrum considered here. For TRA, the following numbers of unique molecular identifiers were used: 13,359 (P. spp., minifish); 1,659,351 (D.r., zebrafish); 570,915 (O.m., rainbow trout); 367,169 (M.m., mouse); 2,654,949 (L.a., elephant). For TRB, the following numbers of unique molecular identifiers were used: 11,215 (P. spp., minifish); 1,300,957 (D.r., zebrafish); 490,529 (O.m., rainbow trout); 1,054,472 (M.m., mouse); 3,511,965 (L.a., elephant). For human sequences (H.s.), the data in Zvyagin et al. [97] were used; from this dataset we extracted 1,766,217 unique TRA and 2,252,311 unique TRB sequences.

One might ask whether the total entropy of antigen receptor repertoires scales with body mass. This seems to be the case, as shown in Figure 11. However, we note that for this analysis to be meaningful, it must take into account the different recombination strategies. That is, such comparisons must be done among species in a similar clade, such as teleosts or mammals. It becomes apparent that irrespective of body size, the total entropy of TCRαβ repertoires is greater in mammalian species than in teleost. Yet, species of similar body mass (and presumably also of similar numbers of lymphocytes that are deployed for immune surveillance and maintenance of tissue homeostasis), thrive in their respective habitat. This raises the interesting question of the minimum diversity of, say, TCRαβ repertoires, that is necessary for a functionally robust immune system. Studies in a SCID‐X1 patient with somatic reversion indicated that a TCRβ repertoire diversity of about 1/10 of healthy controls provided long‐term repertoire stability and protective and regulated T‐cell immunity for at least two decades [98]. Studies of more such patients will be required to assess the role of private versus public components of the minimal repertoires. Judging from our work in minifish [80], it is reasonable to assume that, in such cases, the public fraction of the repertoire provides the required stability. Indeed, in the SCID‐X1 patient, although dramatic expansions and contractions of individual clonotypes representing up to 30% of the repertoire were recorded during the observation period, stable diversity indices revealed that these transient clonal distortions did not cause long‐term repertoire imbalance [98]. Such studies begin to define the minimal amount of TCR repertoire reconstitution that is required after iatrogenic interventions, such as bone marrow transplantation to achieve immune homeostasis.

Another unexplored aspect of the differences in repertoire structure among teleosts and mammals concerns the observation that, in mouse and human, immune responses to antigens have been shown to be largely private [99], with each individual summoning unique CDR3 sequence(s) to drive the response. It will be interesting to explore whether the same is true for teleosts or whether their immune response to pathogens is driven more by the public, possibly cross‐reactive fraction of the repertoire.

## Conflicts of Interest

The authors declare no conflicts of interest.

## Data Availability

Data sharing not applicable to this article as no datasets were generated or analyzed during the current study.
